# Genomic analysis of *Neisseria gonorrhoeae* strains from disseminated, urogenital, and rectal infections reveals differences in genes for iron acquisition and type IV secretion

**DOI:** 10.1128/spectrum.01338-26

**Published:** 2026-08-10

**Authors:** Natalie S. Nunnally, Julie L. Stoudenmire, Odile B. Harrison, Anastasia Unitt, Ellen N. Kersh, Cynthia Nau Cornelissen

**Affiliations:** 1 Institute for Biomedical Sciences, Georgia State University 439338, Atlanta, Georgia, USA; 2 Nuffield Department of Population Health, University of Oxford 227951, Oxford, United Kingdom; 3 Georgia Public Health Laboratory, Georgia Department of Public Health 37380, Decatur, Georgia, USA; LSU Health Shreveport, Shreveport, Louisiana, USA

**Keywords:** *Neisseria gonorrhoeae*, disseminated gonococcal infection, type IV secretion system, gonococcal genetic island, TonB-dependent transporters

## Abstract

*Neisseria gonorrhoeae*, an obligate human pathogen, causes the sexually transmitted infection gonorrhea. Delayed treatment or immunodeficiencies can result in a higher risk of disseminated gonococcal infection (DGI), where the infection spreads to normally sterile anatomic sites, including the bloodstream. The gonococcus produces TonB-dependent transporters (TdTs) to sequester metals from host nutritional immunity proteins. These transporters are critical for survival and are important virulence factors. We characterized genes encoding iron-regulated TdTs in the genomes of strains from disseminated (*n* = 47), urogenital (*n* = 86), and rectal (*n* = 12) infections. We found that gonococcal strains isolated from DGI infections were more likely to express a functional hemoglobin-iron utilization operon (*hpuAB* wild type and phase on [32%, *n* = 15/47]), to harbor nonsense mutations in *tdfF* (57%, *n* = 27/47), and were predicted to express low levels of *fetA* (43%, *n* = 20/47) when compared to strains from urogenital or rectal infections. In contrast, gonococcal strains from localized urogenital infections were associated with a higher frequency of functional *tdfF* genes (66%, *n* = 57/86) and high *fetA* expression (30%, *n* = 26/86). Additionally, strains from rectal infections were more frequently found to have high *fetA* expression (33%, *n* = 4/12) and a functional *tdfF* (75%, *n* = 9/12). Furthermore, we identified associations between the presence and absence of *tdfF* and the gonococcal genetic island, which encodes a type IV secretion system. These findings inform our evolving understanding of the molecular mechanisms underlying disseminated gonococcal infections.

**IMPORTANCE:**

Gonorrhea is an emerging, global health threat, with over 100 million new cases each year. Delayed treatment, immunosuppressive medications, and immunodeficiencies can contribute to the spread of infection to the blood, presenting as a serious disseminated manifestation. With rising antimicrobial resistance and no licensed preventative vaccine, untreatable gonorrhea is a possibility in the near future. TonB-dependent transporters are highly conserved and vital for metal acquisition and survival, making them promising targets for therapeutic or preventative strategies. Associated surface-exposed lipoproteins display greater sequence variation but contribute to metal acquisition. We analyzed the genes encoding TonB-dependent transporters and associated lipoproteins from strains associated with distinct disease manifestations. We conclude that gonococci isolated from disseminated, urogenital, and rectal sites demonstrate different phase and allelic variations in genes encoding iron transport systems and the gonococcal genetic island.

## INTRODUCTION

*Neisseria gonorrhoeae* (*Ngo*), an obligate human pathogen that causes gonorrhea, is an emerging public health threat. The World Health Organization estimated 100 million new cases worldwide in 2020 (1). Furthermore, the increasing incidence of drug resistance prompted the removal of azithromycin from recommended treatments by the Centers for Disease Control and Prevention (CDC) in 2020 (2). Ceftriaxone was until recently the only antibiotic recommended for empiric therapy for uncomplicated urogenital and rectal infections in the United States. Other treatments are now available (2), including new first-in-class antibiotics, gepotidacin and zoliflodacin. Both are effective against *Ngo* and were approved by the FDA in 2025 (3–5). However, resistance to drugs predating these new ones has spread through the population. Thus, resistance has been a persistent issue with *Ngo*, making this pathogen an urgent public health concern (6–8). This is particularly problematic as there currently is no licensed, effective vaccine available for gonorrhea prevention. Recently, a *Neisseria meningitidis* (*Nme*) vaccine, 4CMenB, was recommended in the United Kingdom for the prevention of gonorrhea in high-risk individuals due to partial cross-protection (9, 10). *Ngo* vaccine development has proven challenging due to the ability of the organism to present a highly variable surface structure to the human host. A better understanding of gonococcal pathogenesis could therefore inform the selection of antigens for vaccine formulations and/or the development of new therapies (11).

Urogenital gonococcal infections typically present as urethritis and/or cervicitis. Delayed diagnosis, host immunodeficiencies, and concomitant treatment with complement-inhibiting drugs can contribute to the progression of the infection to the upper reproductive tract or its spread to distant body sites, such as the bloodstream, meninges, heart, and synovial fluid (12, 13). Systemic spread occurs when *Ngo* invades epithelial tissues and enters the bloodstream via unknown mechanisms. Although disseminated gonococcal infections (DGI) are relatively rare, recent increases in incidence (from 2013 to present) are a concern and have prompted further investigation into the genetic basis behind this upsurge (14).

Asymptomatic genital infections are more common in women (15) and can facilitate gonococcal persistence, ascension to the upper genital tract, and dissemination (16–18). Disseminated cases have been historically linked to the onset of menstruation, pregnancy, and complement deficiencies (19). The endometrium displays greater susceptibility to disseminated infections during pregnancy and menstruation due to changes in cervical mucus, vaginal pH, and potentially menstrual blood (20). The administration of complement inhibitors, such as eculizumab, has also been implicated in gonococcal dissemination (21).

Despite historical precedent, recent DGI cases have been reported to be equally distributed between men and women (22). For example, a cluster of genetically linked cases of DGI occurred in Michigan, USA, in 2019, where 56% (*n* = 9/16) of the patients were male. The investigation revealed that 81% (*n* = 13/16) of the patients had the initial manifestation of septic arthritis (23). In 2020, the California Department of Public Health reported a surge in DGI cases, highly associated with primary urogenital, rectal, and pharyngeal infections that were asymptomatic (24). Moreover, the Alaska Section of Epidemiology reported a 10-fold increase in DGI cases between 2022 and 2024, with the majority of patients requiring hospitalization (25, 26). Three investigations in Manitoba, Canada (Public Health Agency of Canada), Georgia, USA (CDC), and Queensland, Australia (Queensland Health) revealed a correlation between the *porB1A* allele in 89% (*n* = 104/116), 67% (*n* = 20/30), and 92% (*n* = 59/64) of DGI cases, respectively (14, 21, 27). The CDC authors also concluded that their DGI isolate collection belonged to multiple genome lineages, and a sub-population of DGI genomes with *porB1B* alleles could also resist complement and survive in human serum (14). PorB1A is an outer membrane porin protein linked to stable complement resistance and increased survival in human serum.

Recently, a multi-state outbreak of *Nme* in the US, involving a urogenital *Nme* clade, demonstrated the significance of genetic diversity, horizontal gene exchange, and niche adaptation. These *Nme* isolates are hypothesized to have acquired the ability to occupy a different niche via DNA acquisition of *Ngo* genes associated with anaerobic respiration and loss of the locus that contains polysaccharide capsule biosynthesis genes (28). *Ngo* and *Nme* are closely related pathogens that typically inhabit distinct niches but can coincide clinically in the bloodstream, urogenital tract, rectum, and oropharynx, providing opportunities for interspecies horizontal gene transfer (29). This observation was the genesis of the current study. We hypothesized that *Ngo* may have acquired *Nme* alleles, thereby increasing the incidence of DGI.

We investigated *hmbR* (cryptic in *Ngo*) and capsule biosynthesis genes (absent in *Ngo*) to determine if the DGI population had evolved to become *Nme*-like. Our analyses did not reveal any apparent genetic resemblance of DGI strains to *Nme* strains (data not shown). Therefore, we focused on other alleles, as described below.

Phase variation of outer membrane structures impacts bacterial fitness. For instance, the two-component hemoglobin-iron acquisition system, comprised of HpuA and HpuB, is phase variable in both *Nme* and *Ngo* (30, 31). HpuAB variation has been linked to fitness in the female genital tract. Variants that produce this system and thereby can use hemoglobin as an iron source are likely selected for when hemoglobin is present in menstrual blood (20). Evolutionary adaptation is closely linked to genetic variation and null mutations in bacteria, which can enhance fitness in novel environments (32).

Most bacteria require iron for survival and virulence (33, 34). As pathogens invade the host, high-affinity innate immunity proteins restrict the uptake of trace metals in a process called nutritional immunity (35). In response, many bacterial species produce and secrete siderophores to remove iron from host metal scavenging proteins. Although *Ngo* does not produce siderophores, it can utilize siderophores synthesized by other bacteria (36–38). *Ngo* obtains iron from xenosiderophores, including enterobactin and salmochelin, via a TonB-dependent transporter (TdT), FetA (36, 39). The gene *tdfF* encodes a TdT that shares 19% sequence identity with FetA and is found exclusively in *Ngo* and *Nme* but not in commensal *Neisseriae*. TdfF is required for intracellular survival in human cervical epithelial cells and is expressed in an iron-dependent manner (40). Additionally, the gonococcal genetic island (GGI), which encodes a type IV secretion system (T4SS), has been shown to compensate for the lack of TdfF and TonB in intracellular survival (41).

Previous publications have analyzed phase-variable genes such as Lipooligosaccharide and complement resistance due to porin type in correlation with DGI presentation (14, 42, 43). Thus, we focused this study on other systems that could contribute to niche-specific iron utilization, some of which are phase variable. Here, we characterized genes encoding iron-regulated TdTs from gonococcal strains associated with DGI and compared them with strains isolated from urogenital and rectal infections in the USA and Canada between 2013 and 2020 (14, 21). We hypothesized that, given the essential role of iron metabolism in invasive disease, DGI, urogenital, and rectal gonococci could possess distinct TdT genotypic profiles that facilitate niche adaptation. We found a correlation between iron acquisition genetic profiles and disease manifestation*,* as well as niche-specific associations between *tdfF* and the GGI, which encodes a T4SS. Comparing the genetic profiles of DGI strains with those of urogenital and rectal infection strains increases our understanding of the molecular basis of distinct gonococcal infections.

## RESULTS

### Identification of distinct *tdfF* alleles among WHO reference *Ngo* strains

**Fig 1:**
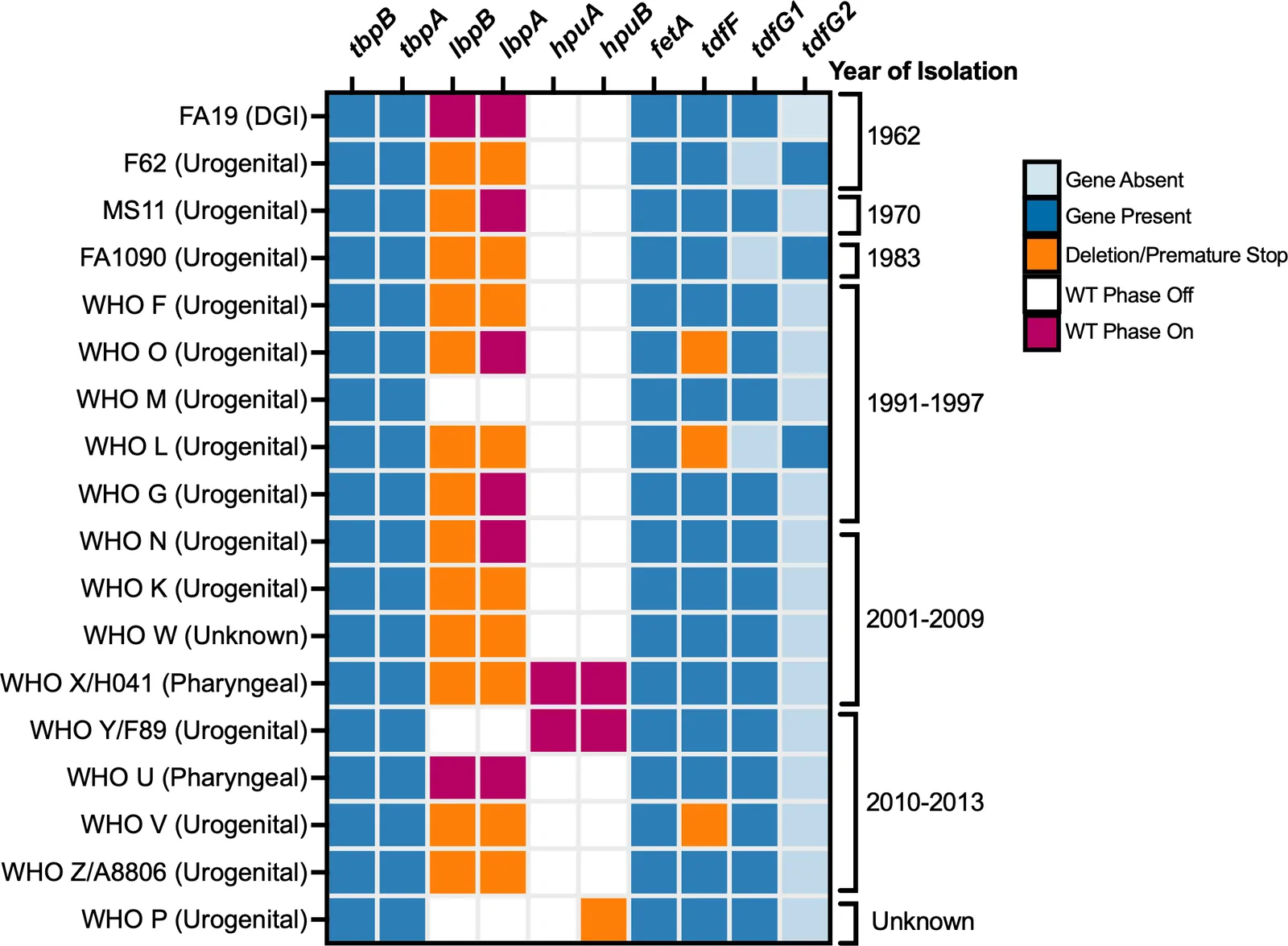
Analysis of genes that encode iron-regulated TonB-dependent transporters and associated surface lipoproteins among common laboratory strains and WHO strains of *Ngo*. Left: strain name (site of infection), top: gene names, and right: year of isolation. There is no slipped strand in the *fetA* structural gene, only in the promoter.

To better understand the distribution of genes encoding iron-regulated TdTs, we analyzed gonococcal whole-genome sequences from a set of laboratory and reference strains (Table 1). Figure 1 shows a heatmap that compares the distribution of gene variants encoding the iron-regulated TdTs and associated surface lipoproteins. The strains were arranged chronologically by known year of isolation and manifestation (DGI, urogenital, or rectal). We detected *tdfF* deletion mutations in three WHO reference strains (WHO O, WHO L, and WHO V [17%, *n* = 3/18]). We translated the nucleotide sequences into deduced amino acid sequences and predicted that these deletions would lead to premature internal stop codons and thereby a truncated, non-functional TdfF.

**TABLE 1:** Demographics of commonly used laboratory strains and recently isolated strains of *Ngo*

Strain	Alternate strain name	Year isolated	Location	Sex	Source	Reference
FA19	–^*a*^	1961	Copenhagen, Denmark	Not found	DGI	(44)
FA1090	–	1983	North Carolina, USA	Female	Endocervical and DGI	(45)
F62	–	1962	Georgia, USA	Male	Urethral	(46)
MS11	–	1970	North Carolina, USA	Male	Urethral	(45)
WHO F	NCTC 13477	1991	Canada	Not found	Urogenital	(47, 48)
WHO G	NCTC 13478	1997	Thailand	Not found	Urogenital	(47, 48)
WHO K	NCTC 13479	2003	Japan	Not found	Urogenital	(47, 48)
WHO L	NCTC 13480	1996	Asia	Not found	Urogenital	(47, 48)
WHO M	NCTC 13481	1992	Philippines	Not found	Urogenital	(47, 48)
WHO N	NCTC 13482	2001	Australia	Not found	Urogenital	(47, 48)
WHO O	NCTC 13483	1991	Canada	Not found	Urogenital	(47, 48)
WHO *P*	NCTC 13484	Not found	USA	Not found	Urogenital	(47–49)
WHO U	NCTC 13817	2011	Sweden	Female	Pharyngeal	(47)
WHO V	NCTC 13818	2012	Sweden	Male	Urethral	(47)
WHO W	NCTC 13819	2007	Hong Kong	Not found	Not found	(47)
WHO X	H041/NCTC 13820	2009	Japan	Female	Pharyngeal	(47)
WHO Y	F89/NCTC 13821	2010	France	Male	Urethral	(47)
WHO Z	A8806/NCTC 13822	2013	Australia	Female	Genital swab	(47)

^*a*^ –, not available.

### Niche-specific differences observed in iron acquisition genes

**Fig 2:**
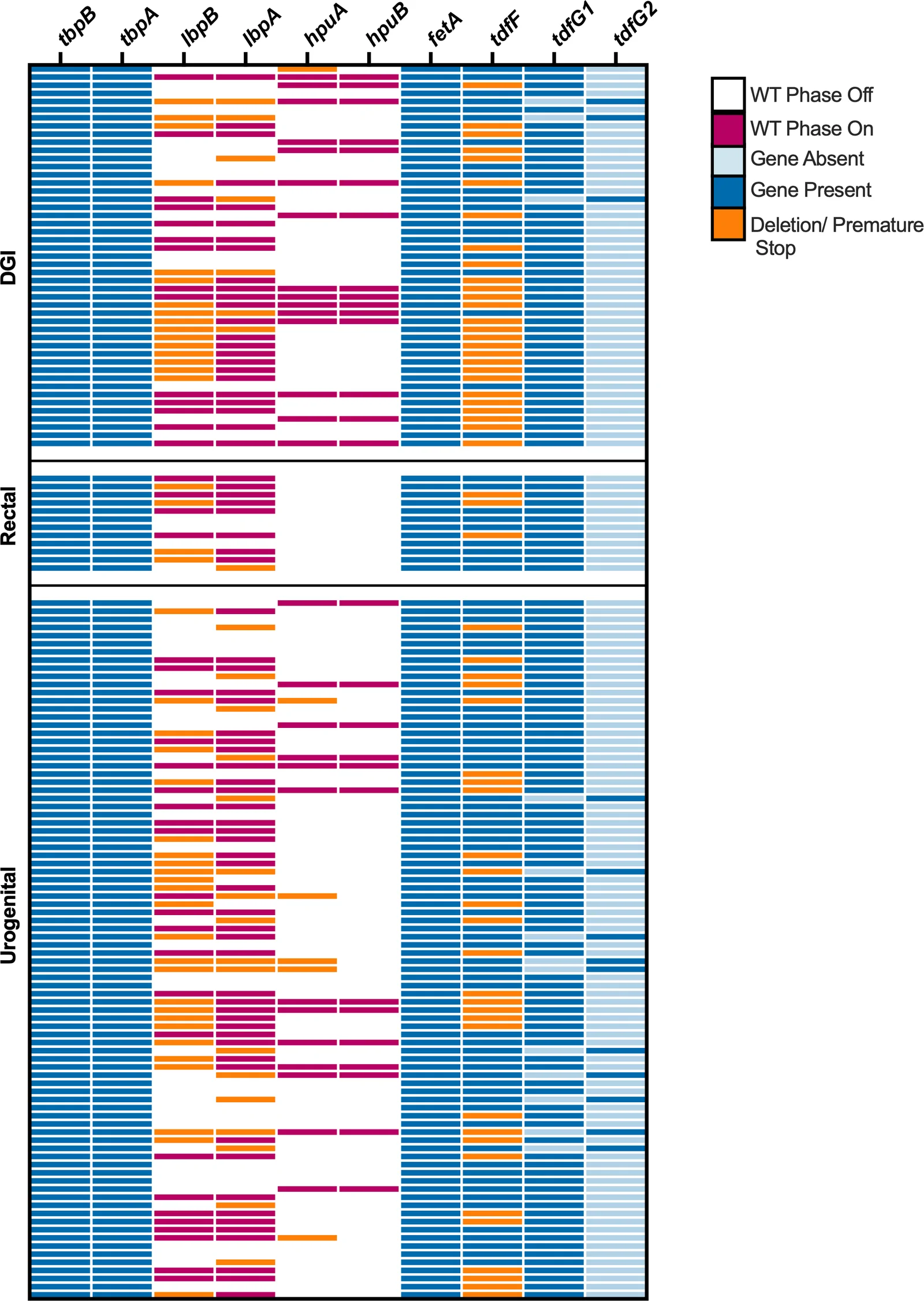
Heatmap distribution of genes encoding iron-regulated TonB-dependent transporters and associated surface lipoproteins in contemporaneously isolated DGI, rectal, and urogenital strains. Left: site of infection, top: gene names, each row represents one isolate. There is no slipped strand in the *fetA* structural gene, only in the promoter.

Next, we investigated the presence or absence of genes encoding iron acquisition systems in whole-genome sequence data spanning 2013–2020. Our genome sequence data set consisted of gonococci associated with DGI (*n* = 47), urogenital (*n* = 86), or rectal (*n* = 12) infections isolated in Georgia, USA, and Manitoba, Canada. We identified nucleotide sequences for genes encoding TdTs implicated in iron acquisition and associated surface lipoproteins. We performed multi-sequence alignments to evaluate niche-specific genetic diversity (Fig. 2). Although a few nonsense mutations were observed in *tbpA* (NEIS1690/NGO1495) and *fetA* (NEIS1963/NGO2093), PCR amplification and subsequent Sanger sequencing confirmed that these apparent mutations were actually sequencing errors (data not shown).

**Fig 3:**
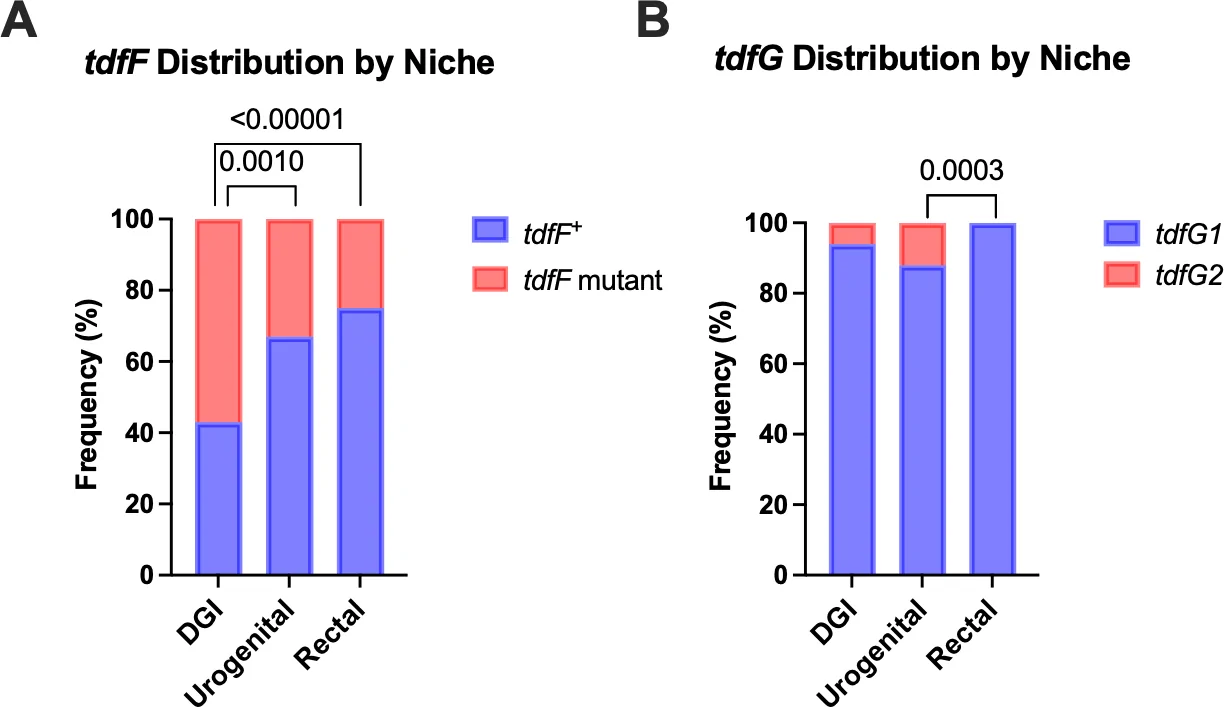
Distribution of *tdfF* and *tdfG* genes by niche. (**A**) Statistical analysis of the presence or absence of *tdfF*. (**B**) Statistical analysis of the presence or absence of two clades of *tdfG* (*tdfG1* and *tdfG2*). A Bonferroni correction was used to determine significance at a threshold of 0.0166.

The *tdfF* (NEIS0338/NGO0021) gene is iron-repressed and encodes a protein critical for survival in cervical epithelial cells under iron starvation conditions (40). The addition of free iron rescued the growth defect of the *tdfF* mutant (40). We detected multiple indels in *tdfF* alleles resulting in deletion mutations, with these most frequently being associated with DGI *Ngo* strains (57%, *n* = 27/47). In contrast, functional *tdfF^+^* were more prevalent in the genomes of urogenital (66%, *n* = 57/86) and rectal (75%, *n* = 9/12) gonococcal strains (Fig. 3A).

Two distinct *tdfG* (NGO0553) variants (denoted as *tdfG1* [NEIS2744] and *tdfG2* [NEIS2646]) share only 59% nucleotide sequence identity, although chromosomal gene synteny is identical. A 1-nucleotide difference in the Fur boxes upstream of *tdfG1* and *tdfG2* may suggest subtle differences in iron regulation (50). Gonococci can possess either *tdfG* variant, with most strains in our collection possessing *tdfG1*. Specifically, the *tdfG1* variant was observed in 94% (*n* = 44/47) of DGI strains, 88% (*n* = 76/86) of urogenital strains, and 100% (*n* = 12/12) of rectal strains. Only 9% (*n* = 13/145) of the strains in this entire analysis had the *tdfG2* gene (Fig. 3B). A previous TdfG protein study utilized a clade-specific antibody for TdfG2 (from strain FA1090) and found that only 17% of *Ngo* strains produced TdfG2 (51), consistent with our broader genomic analysis.

### Contribution of phase variation to niche-adaptive iron acquisition

Some genes encoding surface-exposed proteins can undergo phase variation, which enhances immune evasion and niche adaptation (52, 53). *Ngo* exhibits phase variation via slipped-strand mispairing, in which the DNA polymerase slips on a string of nucleotide repeats during replication (54). Slippage can then result in an increase or decrease in the number of repeat units in the newly synthesized gene. Changes in the number of nucleotide repeat units can occur in the coding region, leading to a frameshift mutation, or in the promoter region, which modulates transcriptional activity (54). The *fetA*, *lbpB*, and *hpuBA* loci are all subject to slipped-strand mispairing and, as a result, display phase variation (30, 54–56).

**Fig 4:**
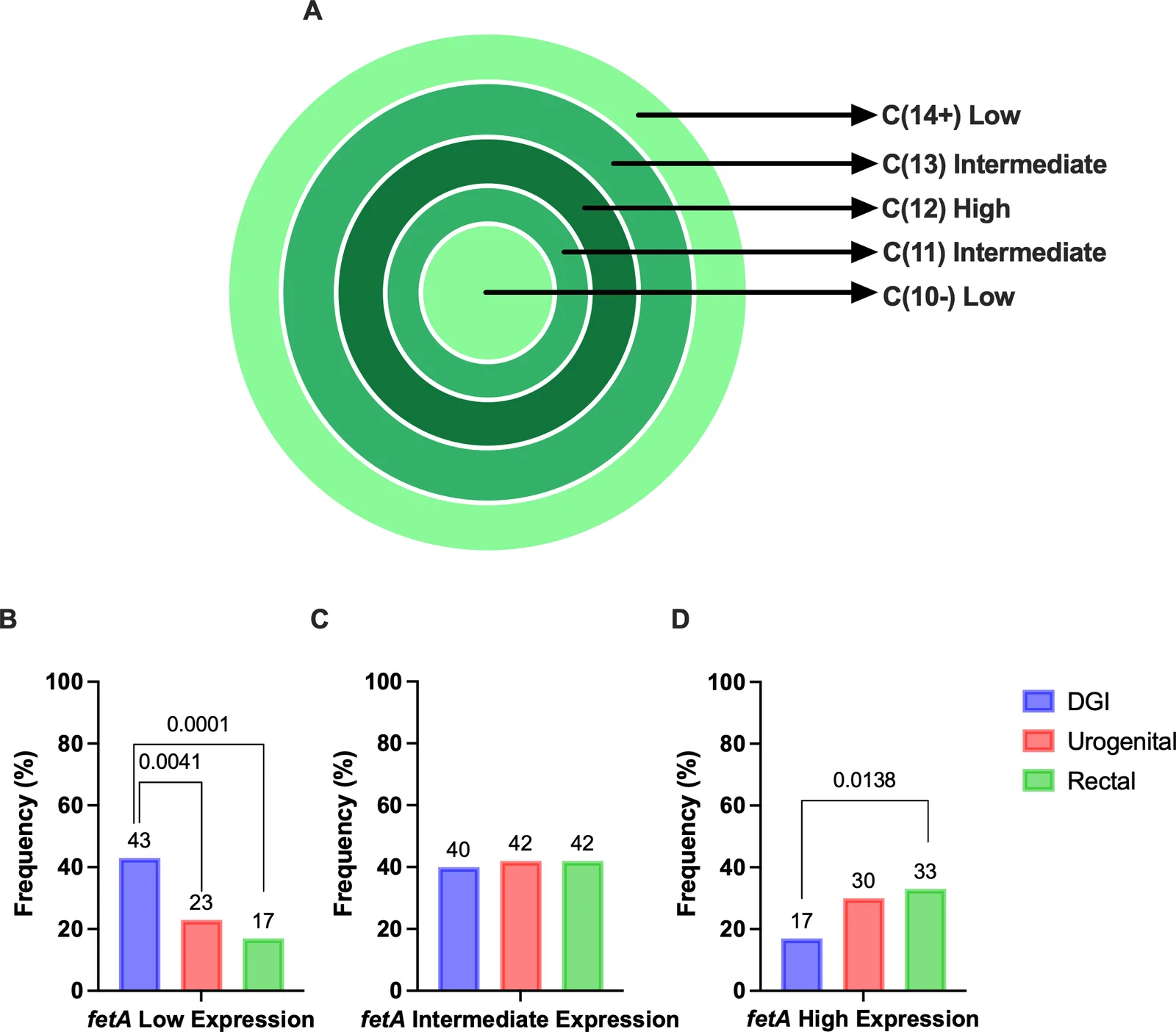
Predicted phase variation in the *fetA* promoter. (**A**) Correlation between the number of C residues in *fetA* promoter and predicted expression levels. Statistical analysis of predicted (**B**) low, (**C**) intermediate, and (**D**) high expression from the *fetA* promoter based on the number of nucleotides in the poly-C tract in the different niches. The numbers represent percent frequencies within each niche population. A Bonferroni correction was used to determine significance at a threshold of 0.0166.

A poly-cytosine (poly-C) tract is upstream of *fetA* between the −35 and −10 consensus sequence of the promoter; changes in the number of C residues due to slipped-strand mispairing result in modulated *fetA* transcript levels (54). *fetA*, which encodes the xenosiderophore receptor, is expressed at the highest level when the poly-C tract contains 12 residues. When the poly-C tract has 11 or 13 nucleotides, *fetA* is expressed at intermediate levels, and any number of nucleotides less than 11 or more than 13 correlates with low gene expression (57) (Fig. 4A). The DGI strains from this analysis showed a higher frequency (43%, *n* = 20/47) of low-expressing *fetA* alleles (Fig. 4B) than in the other two populations; high *fetA* expression strains represented only 17% (*n* = 8/47) of the DGI strain population. There were no statistical differences in strains producing intermediate levels of *fetA* (Fig. 4C). By contrast, alleles corresponding to high *fetA* expression were detected more frequently in rectal (33%, *n* = 4/12) and urogenital (30%, *n* = 26/86) strains (Fig. 4D).

**Fig 5:**
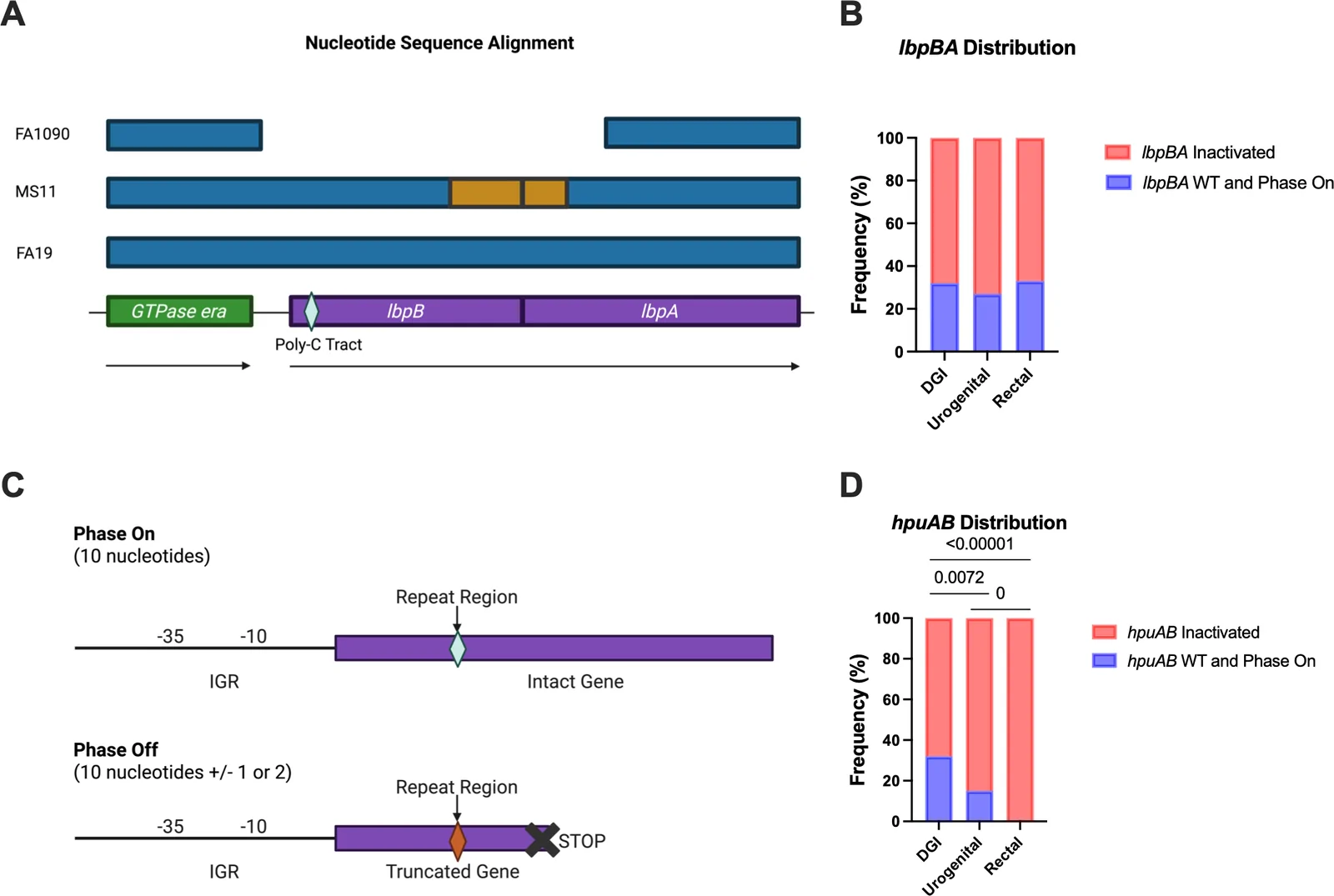
Predicted phase variation within the *lbpBA* and *hpuAB* iron acquisition systems. (**A**) A depiction of the sequence diversity in *lbpBA* operon in three type strains of *Ngo*. The gold-highlighted region in MS11 represents a region of sequence diversity compared to strain FA19. Below FA19 is a schematic of the locus, including the upstream GTPase-encoding gene *era*. The poly-C tract is shown in the *lbpB* gene. Panel A was created with BioRender. (**B**) Distribution of strains with a WT and phase on vs inactivated *lbpBA* system. (**C**) Schematic showing the mechanism of phase variation for the *hpuAB* iron acquisition system. (**D**) Distribution of strains with a WT and phase on vs inactivated *hpuAB* system. IGR stands for intergenic region. A Bonferroni correction was used to determine significance at a threshold of 0.0166.

*lbpB* (NEIS1469) encodes a surface-exposed lipoprotein that enhances human lactoferrin-iron uptake. A poly-C tract is located within the coding region of *lbpB*, which lies upstream of the *lbpA* gene encoding the TdT. The slipped strand repeat region in the *lbpB* gene acts as an on/off phase variation switch for the two-gene operon (55). *lbpB* is phase on when 10 nucleotides are present. When there are 10 nucleotides ± 1 or 2, a frameshift mutation occurs, turning off both *lbpB* and, due to polarity, *lbpA* (Fig. 5A). Type strains FA1090, MS11, and FA19 possess three distinct *lbpBA* loci (Fig. 5A). The locus in FA19 is fully wild type, whereas the locus in FA1090 is deleted so that both genes are inactivated. The locus in MS11 contains multiple mutations relative to FA19, such that the *lbpB* gene is truncated and the *lbpA* gene is functional. This operon produces a functional lactoferrin utilization system (58). Only a small sub-population of strains in our study were found to have a wild-type and phase on *lbpBA* operon (DGI, 32%, *n* = 15/47; urogenital, 27%, *n* = 23/86; rectal, 33%, *n* = 4/12; Fig. 5B).

The *hpuA* gene encodes a surface-exposed lipoprotein, which is phase variable (30, 31). A poly-guanine (poly-G) tract is located at the 5′ end of *hpuA* and serves as an on/off phase variation switch. When 10 nucleotides are present, *hpuA* is phase on. A frameshift mutation occurs when there are 10 residues ± 1 or 2, which turns the gene off (Fig. 5C). The majority of gonococcal strains in this study harbored mutations in the *hpuA* (NEIS1946/NGO11390) gene and were therefore functionally off (81%, *n* = 117/145; Fig. 5D). Nevertheless, the two-gene operon, *hpuAB*, was wild type and phase on most frequently (32%, *n* = 15/47) in DGI strains, compared to urogenital (15%, *n* = 13/86) and rectal strains (0%, *n* = 0/12) in our data set (Fig. 5D).

### Population structure of DGI, urogenital, and rectal *Ngo*

**Fig 6:**
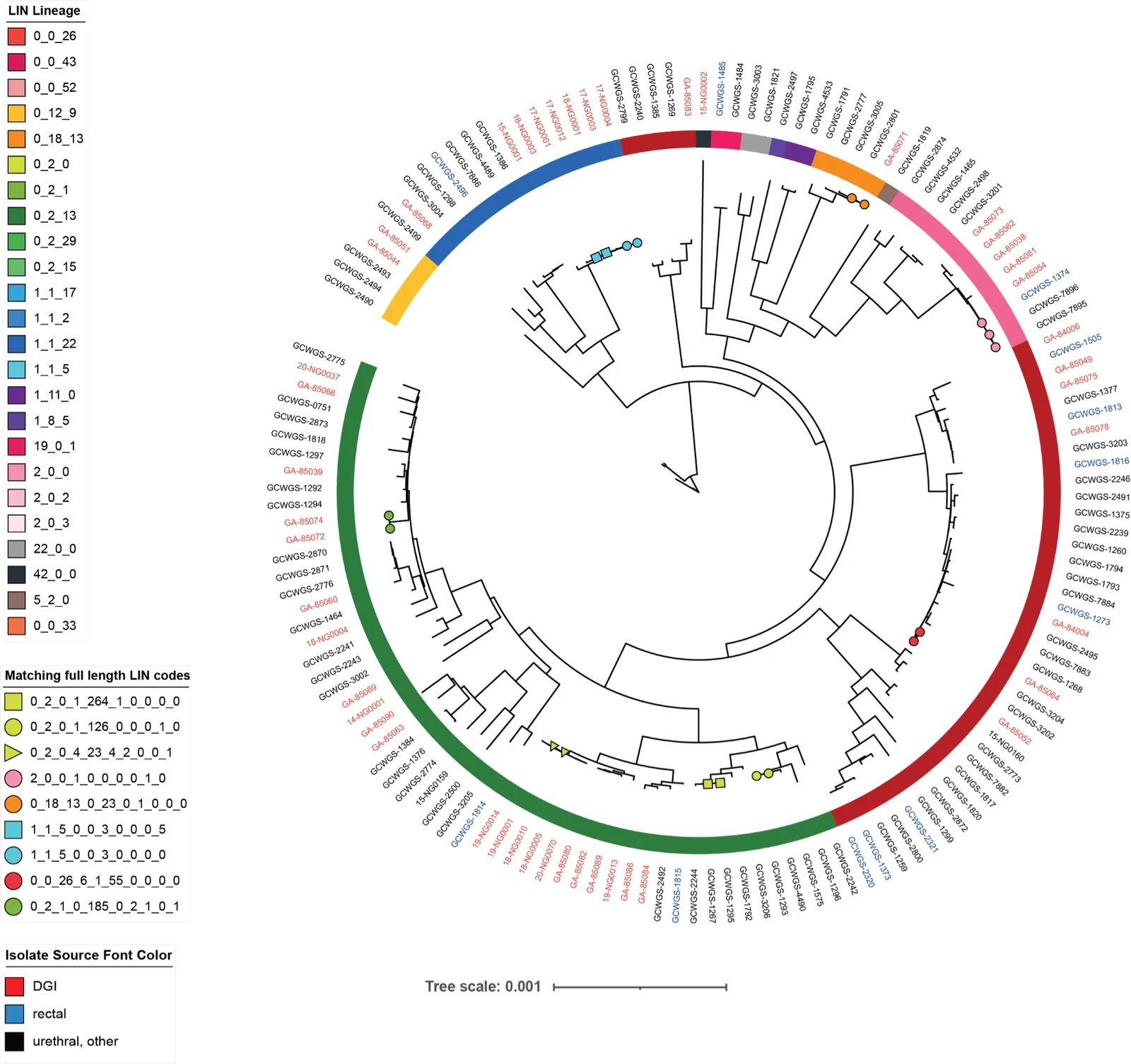
Population structure of DGI, urogenital, and rectal *Neisseria gonorrhoeae* from this study. Maximum likelihood tree constructed using 1,430 loci from Ng cgMLST v2. The inner ring displays the Life Identification Number (LIN) codes by color. Gonococcal strains with matching LIN codes were labeled on the tree nodes. The strains are differentiated by font color according to source of isolation (DGI, red; rectal, blue; urogenital/urethral, black).

We evaluated DGI, urogenital, and rectal gonococcal lineages using Ng cgMLST v2. This scheme within the PubMLST database comprises 1,430 core gonococcal loci that remain stable over time, along with Life Identification Number (LIN) codes, which have been shown previously to provide a stable form of nomenclature. LIN codes offer information on hierarchical clustering at successive thresholds within the cgMLST scheme (59). Figure 6 shows a maximum likelihood tree generated from whole-genome sequence alignments, supported by 100% bootstrap values. Strains within our data set that shared an entire LIN code were defined as clonal groups. A total of 19% of DGI (*n* = 9/47), 10% of urogenital (*n* = 9/86), and 8% of rectal (*n* = 1/12) strains in this collection clustered into various clonal groups; however, the majority of the data set harbored unique LIN codes (Fig. 6) and were therefore not clonal, consistent with previous studies (14).

### *tdfF* phylogeny and niche-associated mutations

**Fig 7:**
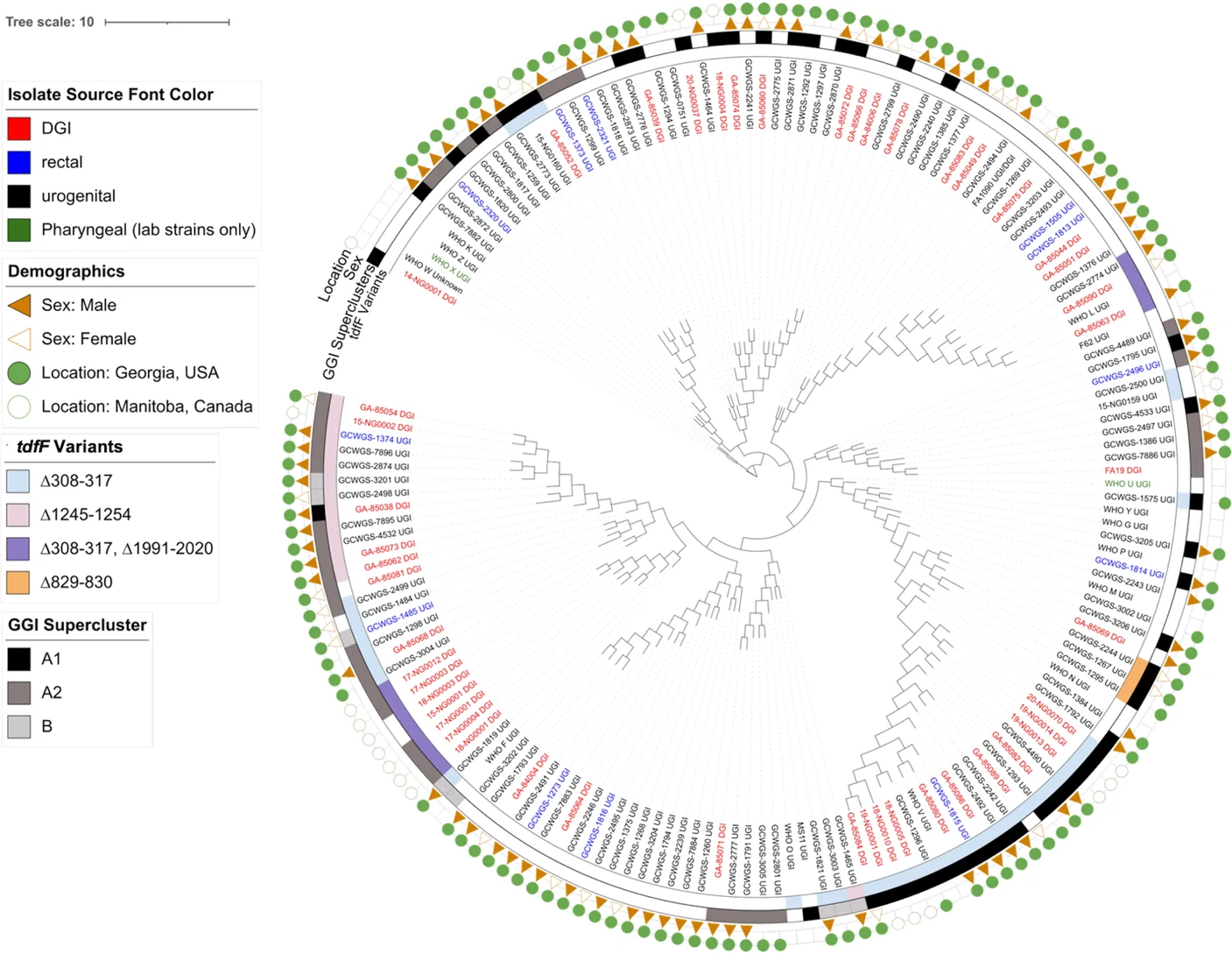
Genetic diversity of *tdfF* deletion mutations in contemporary DGI, urogenital, and rectal gonococcal infection strains. A maximum likelihood tree of *tdfF* nucleotide sequences. Patient demographics are displayed in the outer rings. GGI superclusters are labeled in the third ring in black (A1), dark gray (A2), light gray (B), or white (strains lacking a GGI). *tdfF* variants are labeled in the fourth ring in light blue, pink, purple, and orange. Strains are labeled in the innermost ring according to source of isolation (DGI, red; rectal, blue; urogenital, black; pharyngeal, green). Genetic relatedness of the *tdfF* sequences is shown in the center.

To evaluate the genetic diversity of *tdfF* alleles, *Ngo* genomes from reference and recent clinical isolates from this data set were mapped and aligned to the reference strain FA19. Nucleotide sequences of *tdfF* were extracted, aligned, and analyzed for polymorphisms. Figure 7 shows the maximum likelihood tree generated from *tdfF* nucleotide sequence alignments, supported by 100% bootstrap values. The alignment revealed a high degree of nucleotide sequence identity among *tdfF* alleles from DGI, urogenital, and rectal strains, ranging from 98% to 100%. Although high conservation was observed, some *tdfF* alleles harbored distinct indels, mostly at the 5′ end of the gene, leading to premature stop codons. These indels were randomly dispersed throughout the phylogenetic tree (Table 2, Fig. 7). A total of five *tdfF* indels were identified. The most frequent mutations were *tdfF*_∆308–317_, *tdfF*_∆1,245–1,254_, and the double indel *tdfF*_∆308–317, ∆1,991–2,020_, all of which were primarily observed in DGI strains (57%, *n* = 27/47; Table 2, Fig. 7). We observed only two alleles containing indels *tdfF*_∆1,991–2,020_ (1%, *n* = 1/86) and *tdfF*_∆829–830_ (3%, *n* = 3/86) among the urogenital strains (Table 2, Fig. 7). TdfF amino acid sequences were deduced from nucleotide sequence data to predict the impact of indels on protein production. All *tdfF* indels found, except *tdfF*_∆1,991–2,020_, were predicted to result in truncated TdfF proteins as a result of premature internal stop codons. More than half of the DGI strain population (57%, *n* = 27/47) harbored *tdfF* alleles containing premature stop indels, which is significantly higher than detected among urogenital or rectal strains (Table 2).

**TABLE 2:** Comparison of *tdfF* mutation frequencies by niche population

Mutation	DGI (%, *n*/*N*)	Urogenital (%, *n*/*N*)	Rectal (%, *n*/*N*)
∆308–317	28 (13/47)	20 (17/86)	17 (2/12)
∆1,991–2,020	0 (0/47)	1 (1/86)	0 (0/12)
∆1,245–1,254	11 (5/47)	8 (7/86)	8 (1/12)
∆308–317 and ∆1,991–2,020	19 (9/47)	1 (1/86)	0 (0/12)
∆829–830	0 (0/47)	3 (3/86)	0 (0/12)

### Associations between the presence of the GGI locus and *tdfF* alleles

The GGI encodes a T4SS and has been found in 61%–80% of gonococcal strains, depending on the study (60–63). In our current analysis, we determined that 66% (*n* = 31/47) of the DGI strains possessed the GGI (GGI^+^), with 59% of the urogenital strains being GGI^+^ (*n* = 51/86) and 67% of the rectal strains being GGI^+^ (*n* = 8/12). In previous studies, the GGI was shown to contribute to TonB-independent intracellular survival, metal-ion trafficking, and biofilm formation (41, 63, 64).

**Fig 8:**
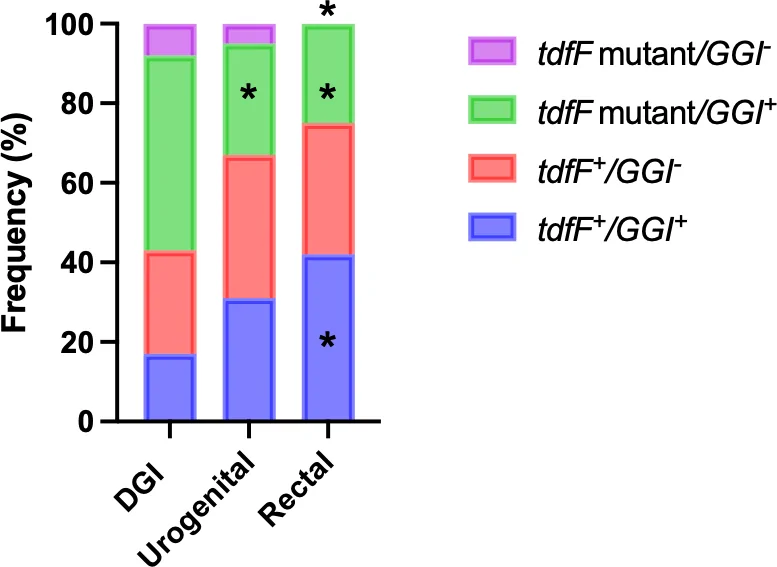
Distribution of the gonococcal genetic island (GGI) in association with *tdfF*. Correlation between the presence and absence of *tdfF* and the GGI. A Bonferroni correction was used to determine significance at a threshold of 0.0166. Asterisks indicate significant differences in pairwise comparisons with the DGI group frequencies. The asterisk above the rectal group indicates that the *tdfF* mutant/GGI^−^ group was significantly different when comparing the DGI niche to the rectal niche. The latter group is not shown in purple because the frequency of occurrence was zero.

Figure 7 shows a maximum likelihood tree of aligned *tdfF* nucleotide sequence data, labeled with the presence/absence of the GGI. Statistically significant associations between *tdfF* and the presence of the GGI were observed (Fig. 8). Co-occurrences of the *tdfF^+^* and GGI^+^ were frequently observed in the rectal gonococcal strains (42%, *n* = 5/12), and this was significantly higher than the DGI (17%, *n* = 8/47) and urogenital (31%, *n* = 27/86) strain populations (Fig. 8). Additionally, the co-occurrence of non-functional *tdfF* alleles and GGI^+^ was frequently observed in DGI gonococci (49%, *n* = 23/47), and this was statistically higher than in urogenital (28%, *n* = 24/86) and rectal (25%, *n* = 3/12) strains (Fig. 8).

### Co-occurrences of GGI superclusters A1 and A2 with *tdfF* alleles

**Fig 9:**
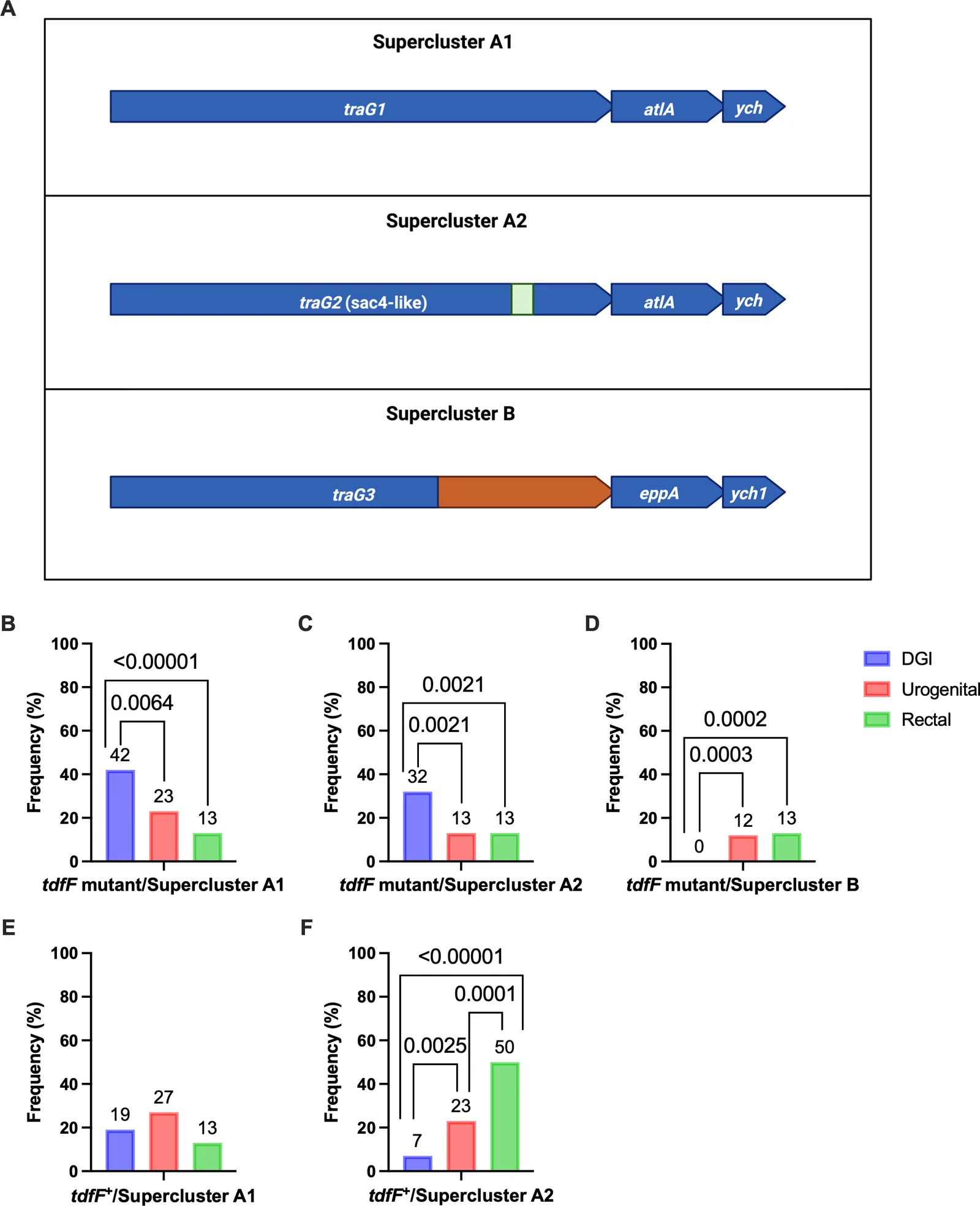
*tdfF* association with GGI superclusters A1, A2, and B. (**A**) Genetic differences between GGI superclusters (65). The green sector in supercluster A2 represents the sac-4-like mutation, and the orange sector in supercluster B represents a genetically distinct region. (**B–F**) Statistical analysis of correlation between *tdfF* and GGI superclusters and niche of isolation. A Bonferroni correction was used to determine significance at a threshold of 0.0166. Corresponding gene name designations: *traG* (NEIS2273), *atlA* (NEIS2274), *ych/ych1* (NEIS2275), and *eppA* (NEIS2311).

The GGI has formerly been divided into three superclusters: A1 (*traG1*, *atlA*, and *ych*) and A2 (*traG2*, sac-4-like, *atlA*, and *ych*), which produce functional DNA secretion systems, and B (*traG3*, *eppA*, and *ych1*), which encodes a T4SS that is non-functional for DNA secretion (Fig. 9A) (65). The majority of DGI (40%, *n* = 19/47) and urogenital (30%, *n* = 26/86) strains in the current data set that were GGI^+^ fell within supercluster A1, which corresponds to the production of a DNA-secreting T4SS (Table 3). Rectal gonococcal strains that were GGI^+^ were mostly categorized as supercluster A2 (42%, *n* = 5/12; Table 3). Only a small portion of urogenital (7%, *n* = 6/86) and rectal (8%, *n* = 1/12) strains belonged to supercluster B (Table 3). Among GGI^+^ DGI strains, those with non-functional *tdfF* alleles were only associated with a T4SS that was capable of DNA secretion, namely superclusters A1 (42%, *n* = 13/31) and A2 (32%, *n* = 10/31; Fig. 9B through F). None of the strains from our study were categorized as having a functional *tdfF* and GGI supercluster B (data not shown).

**TABLE 3:** Frequency of GGI superclusters in DGI, urogenital, and rectal infection strains^*a*^

GGI supercluster	DGI (%, *n*/*N*)	Urogenital (%, *n*/*N*)	Rectal (%, *n*/*N*)
Supercluster A1	40 (19/47)	30 (26/86)	17 (2/12)
Supercluster A2	26 (12/47)	22 (19/86)	42 (5/12)
Supercluster B	0 (0/47)	7 (6/86)	8 (1/12)
GGI^−^	34 (16/47)	40 (34/86)	33 (4/12)

^*a*^ Strains labeled as GGI^−^ do not possess a GGI.

### Sex differences in distribution of genotypes among urogenital strains

Because the urogenital tracts of males and females represent significantly different ecological niches, we compared the genomes from the urogenital isolates that came from men and women. We found that there were statistical differences between these groups in the *tdfF* and *tdfG* genes (Table 4). Specifically, male urethral samples had a higher frequency of intact *tdfF* alleles and a higher percentage of *tdfG2* alleles.

**TABLE 4:** Distribution of genotypes by urogenital male vs female infection strains

	Urogenital—males	Urogenital—females	*P* value
	%, (*n*/*N*)	%, (*n*/*N*)	
*tdfF^+^*	75 (42/56)	45 (5/11)	<0.0001
*tdfF^−^*	25 (14/56)	55 (6/11)	<0.0001
GGI supercluster A1	29 (16/56)	18 (2/11)	ns^*a*^
GGI supercluster A2	20 (11/56)	18 (2/11)	ns
GGI supercluster B	7 (4/56)	9 (1/11)	ns
*tdfG1*	88 (49/56)	100 (11/11)	0.0003
*tdfG2*	12 (7/56)	0 (0/11)	0.0003
*hpuAB* inactivated	91 (51/56)	82 (9/11)	ns
*hpuAB* WT and phase on	9 (5/56)	18 (2/11)	ns
*lbpBA* inactivated	70 (39/56)	82 (9/11)	ns
*lbpBA* WR and phase on	30 (17/56)	18 (2/11)	ns
*fetA* low expression	18 (10/56)	18 (2/11)	ns
*fetA* intermediate expression	50 (28/56)	36 (4/11)	ns
*fetA* high expression	30 (17/56)	36 (4/11)	ns

^*a*^ ns, no significance.

## DISCUSSION

To establish infection, *Ngo* must overcome the host immune response, metal limitations, and environmental stressors (66). Selective pressure drives frameshift mutations in bacteria, thereby promoting host adaptation and immune evasion. Phase variation, facilitated by slipped-strand mispairing, is a common evasion strategy that allows for reversible ON/OFF switching of gene expression (67). *Ngo* employs nutrient acquisition systems comprising TdTs that sequester metals from host proteins, thereby enabling growth (66). With the increasing incidence of DGI, we investigated the allelic diversity of genes encoding iron-regulated TdTs in strains associated with disseminated vs urogenital and rectal infections. Identifying virulence factors associated with DGI could yield important insights into neisserial virulence.

Our analysis identified two *tdfG* variants, designated as *tdfG1* (NEIS2744) and *tdfG2* (NEIS2646), with 59% nucleotide sequence identity. A 1-nucleotide difference in the Fur boxes upstream of *tdfG1* and *tdfG2* suggests a subtle difference in iron regulation, although the synteny around the *tdfG* clades is identical. The majority of strains in our collection possessed *tdfG1*, although this genotype was most prevalent in rectal gonococcal strains. *tdfG* encodes a TdT that currently has no known ligand or biological function. *tdfG2* has been shown to be repressed by Fur, a transcriptional regulator that senses iron limitation (50, 51). The rectum provides hospitable conditions for a variety of commensals and pathogens (68), and TdfG1 may contribute to the survival of *Ngo* in this competitive environment.

The LbpBA acquisition system is not required for infectivity, although it increases competitive fitness for *Ngo* both *in vitro* and *in vivo* (58). LbpA is the TdT that binds to human lactoferrin, which is found in milk, secretions, and neutrophil granules (69). Historically, LbpA has been found in all *Nme* strains and only ~50% of the gonococcal population (55, 58, 70, 71). We observed the wild type and phase on *lbpBA* genotype in ~30% of genomes in this study, suggesting a selection against this system in all three niches. Lactoferrin releases a cationic peptide called lactoferricin during acidic proteolysis, which has bactericidal effects (72). It is conceivable that *Nme* strains may be less susceptible to lactoferricin due to the polysaccharide capsule, which is absent in *Ngo* strains.

**Fig 10:**
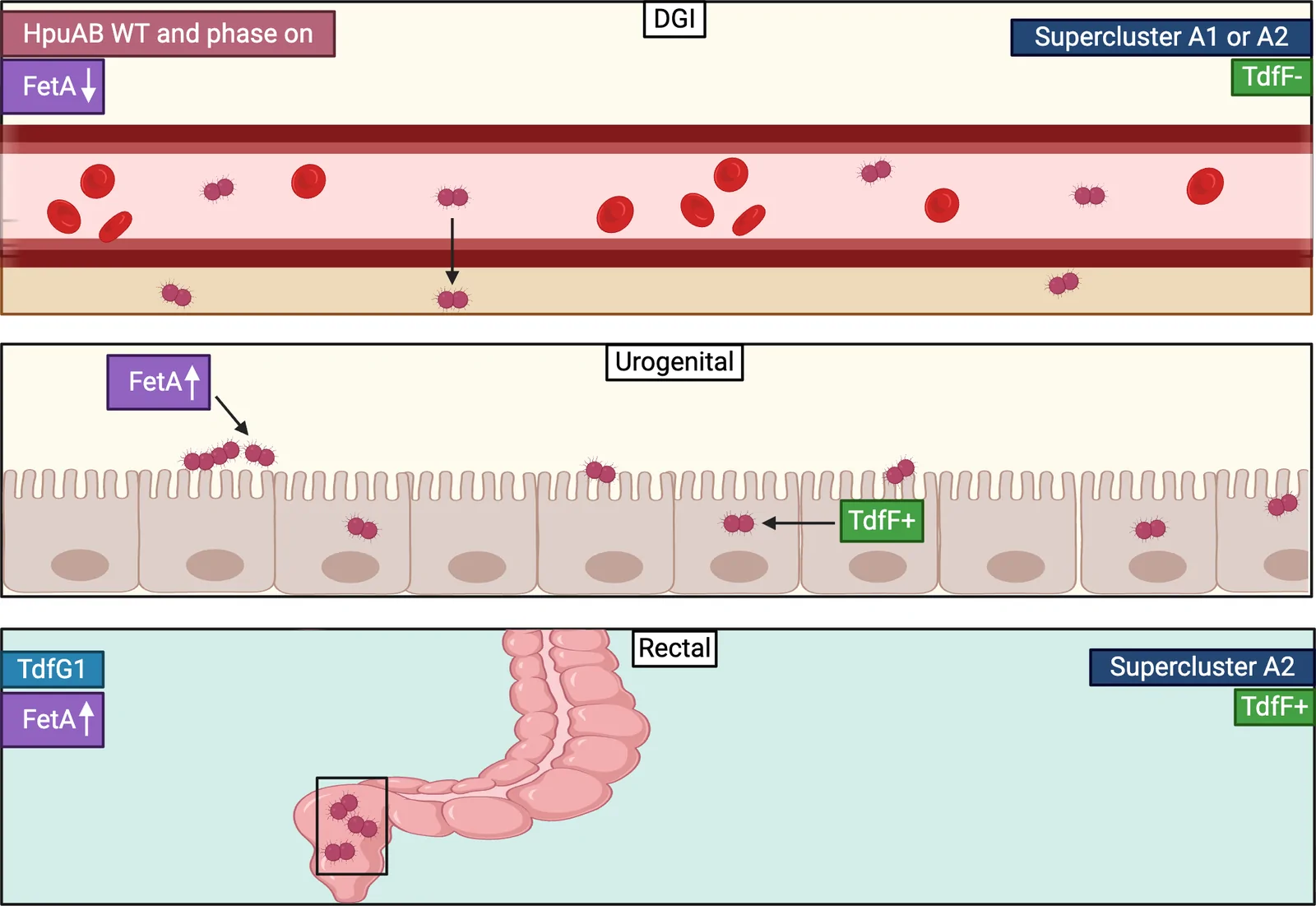
Model of functional iron acquisition systems and *Ngo* disease manifestations. DGI strains (top panel) in this data set were found to have a higher frequency of *hpuA*^+^ and be phase on than urogenital and rectal strains. Also, DGI gonococci were observed to exhibit *tdfF* mutations and alleles corresponding to low expression of FetA more frequently. The inactivation of *tdfF* in this niche was found to correlate with the presence of GGI superclusters A1 and A2. Urogenital strains (middle panel) in this collection had alleles corresponding to higher expression of FetA and were *tdfF^+^* more frequently than those from the other niches. The rectal strains (bottom panel) in this data set were more frequently *lbpA^+^, tdfG1*, and *tdfF ^+^* than disseminated and urogenital strains. Higher predicted expression of FetA was also noted in the rectal strains. Additionally, a correlation between *tdfF^+^* and GGI supercluster A2 was observed more frequently in the rectal strains. Figure was created with BioRender.

The HpuAB system facilitates human hemoglobin-iron uptake, and both proteins are required for hemoglobin binding and iron acquisition (30, 56). Expression of this locus has previously been linked to gonococcal infections in women during the first half of their menstrual cycle (20). This transport system has also been associated with increased virulence in *Nme* (73, 74). In the current study, we observed a similar correlation in disseminated gonococci. DGI strains analyzed here were more frequently *hpuAB* wild type and phase on compared to urogenital and rectal gonococcal strains. However, the majority of strains in this study harbored *hpuA* frameshift mutations. A previous report revealed moonlighting functions for meningococcal *hpuA*, including adhesion and invasion (75). Taken together, our findings suggest that there may be a correlation between HpuA utility and invasive gonococcal disease (Fig. 10).

The xenosiderophore transporter, FetA, is subject to variation that results in adjustable transcription levels, possibly to balance iron acquisition and immune evasion (54). FetA is immunogenic and well conserved among *Neisseriae*, making it a potential vaccine candidate (76–78). In our data set, we frequently observed the genotype associated with low production of FetA in DGI strains compared to rectal and urogenital gonococcal strains (Fig. 10). Our results suggest that *fetA* expression may be increased during localized *Ngo* infections, likely to enable utilization of catecholate siderophores provided by normal flora in the urogenital and rectal niches, thereby supporting growth and infection.

We discovered that *tdfF* alleles contained indels more frequently present in strains associated with DGI. The mutated *tdfF* alleles mostly harbored deletions at the 5′ end of the gene, resulting in amino-terminal truncation of the predicted protein and loss of function. The *tdfF^+^* was more frequently found in urogenital and rectal gonococcal strains. Together, these findings are consistent with positive selection for TdfF functionality in the urogenital and rectal niches. This observation is consistent with the demonstrated role for this protein in intracellular iron acquisition within cervical epithelial cells (40).

This study suggests that distinct iron transport mechanisms may be necessary and differentially selected for in disseminated, urogenital, and rectal gonococcal infections. The GGI encodes a T4SS that secretes DNA (60). Previously, *Ngo* strains with and without the GGI were reported to form sub-populations within the host, allowing them to inhabit different niches. Links have been made between disseminated infections and the GGI, possibly related to biofilm formation and metal ion trafficking. Iron and manganese transport variants have also been shown to be strongly associated with the presence of a GGI (60, 63). Therefore, we investigated whether the GGI was associated with allelic variants of *tdfF*. We identified significant co-occurrence of *tdfF* variant alleles with GGI superclusters A1 and A2 in disseminated strains, both of which encode secretion-competent T4SS. We also observed a correlation of *tdfF^+^* with GGI supercluster A2 in rectal strains. Our analysis suggests that TdfF and GGI supercluster A2 together may provide a benefit to the gonococcus during rectal infections. Furthermore, GGI superclusters A1 and A2 may confer a benefit solely for invasive gonococcal disease. TdfF and the T4SS have compensatory functions contributing to intracellular survival within cervical epithelial cells (40, 41).

This analysis was somewhat limited by the relatively small number of rectal strains in our database. We initially combined data from urogenital and rectal strains as examples of “uncomplicated gonorrhea” (Table S1). The results from this combined analysis with more total strains were not different from the results when the groups were analyzed separately. That is, both the rectal and urogenital isolates had similar, statistically significant differences from the DGI strains in the *hpuAB* and *tdfF* loci. Another potential limitation to our analysis is that variations in some genes that we considered are due to high-frequency phase variation of short repeat regions. It should be noted, however, that statistical differences were detected in both the phase-variable *hpuAB* locus and the *tdfF* locus, which is not phase variable. Thus, the differences detected in this study cannot solely be due to phase variation, which cannot be controlled when strains are passaged in the laboratory prior to sequencing.

While we did not find that *Ngo* strains causing DGI had acquired *Nme*-specific genes that have known contributions to disseminated disease (genes encoding capsule and HmbR), we determined that expression of HpuAB, LbpBA, FetA, TdfG1, and TdfF may be associated with niche adaptation. Genetic variation frequently occurs in genes encoding these nutrient acquisition proteins, potentially providing differential benefits in disseminated, urogenital, and rectal gonococcal infections. Frequent mutations occur in the gene encoding TdfF; these mutated alleles are more commonly identified in disseminated *Ngo* strains with a functional GGI. This study provides the first evidence correlating iron acquisition strategies deployed by *Ngo* with disease manifestation and niche adaptation. Additionally, these findings provide insight into where these potential antigens are expressed within the host. With the increasing incidence of DGI, further research is needed to identify conserved antigens required for severe gonococcal illness.

## MATERIALS AND METHODS

### Isolate selection

In this study, we analyzed three sets of *Ngo* strains that had been previously sequenced and assembled by the Sexually Transmitted Diseases Laboratory Reference and Research Branch at the CDC (14) and the Department of Medical Microbiology and Infectious Diseases at the University of Manitoba (21). Sequence read quality was previously assessed using FastQC Kraken (version 0.10.5) and FastQC Galaxy (version 0.71). High-quality reads were previously assembled using Spades “careful” option (version 3.9.0), Flye (version 2.8), Snippy (version 4.3.8), and Shovill (Galaxy version 1.04 + galaxy0). The first set comprised DGI whole-genome sequences (*n* = 47), including those from *Ngo* strains collected from blood, synovial fluid, or peritoneal fluid. The second set included whole-genome sequences from strains isolated from urogenital sites (*n* = 86; 56 from males, 11 from females, and 19 from people of unknown sex). A third set contained whole-genome sequences from strains isolated from rectal sites (*n* = 12). All strains were isolated in Georgia, USA, or Canada between 2013 and 2020, within the same contemporary time frame (Table S2). Common laboratory strains and geographically diverse reference strains (the WHO collection) were also analyzed (Table 1; Table S2).

### Comparative phylogenetic analysis

Comparative phylogenetic analysis was performed against reference strain *Ngo* FA19 (NCBI accession no. CP012026), which was initially derived from a patient with disseminated gonococcal infection in 1962, as described previously (70). Whole-genome sequences were mapped to nucleotide sequences from FA19 using Geneious Prime version 2023.2.1 (https://www.geneious.com) and aligned with MUSCLE version 5.1. Deletion mutations were annotated, and the nucleotide sequences were translated into deduced amino acid sequences. Frameshift mutations in *fetA*, *tbpA*, and *tdfF* were confirmed by PCR amplification followed by Sanger sequencing of strains within our possession. We utilized Geneious Prime and Microsoft Excel for data analysis.

### Phylogenetic tree analysis

Whole-genome sequences were first aligned with the reference strains, FA19 or MS11, using MUSCLE version 5.1. Maximum likelihood phylogenies were constructed using RAxML using the GTR CAT nucleotide substitution model and a bootstrap analysis based on 1,000 replicates. The trees were visualized and annotated with Interactive Tree of Life (iTOL) (79). A total of 100% bootstrap values supported branch designations.

### Core genome MLST and LIN codes

Ng cgMLST (core genome MLST) and LIN codes were determined using PubMLST, a public database for bacterial genetic analyses (https://pubmlst.org/organisms/neisseria-spp). In this work, we applied Ng cgMLST version 2, comprising 1,430 core genes (59). These core genes are applied in a hierarchical clustering approach to define LIN codes, which numerically encode an isolate’s cluster number at sequential threshold levels of allelic differences (80). Gonococcal LIN codes are assigned by uploading a whole-genome sequence to PubMLST, where the core genome is then compared against isolates already in the database to assign appropriate cluster numbers. The whole-genome sequence data applied in this study, and corresponding LIN codes, will be freely available on PubMLST following publication (Table S2). The maximum likelihood tree was generated in iTOL, and 100% bootstrap values supported the branch designations.

### Determining GGI presence and absence

We used PubMLST to determine the presence or absence of the GGI within our strain collection (81). Whole genomes were mapped to GGI genes from reference strain MS11 (GenBank accession no. CP003909.1) for downstream sequence analysis.

### Statistics

Fisher’s exact test was used to determine whether associations between disseminated, urogenital, and rectal strains were statistically significant. *P* values of <0.05 were considered significant. A Bonferroni correction was used to further assess significance.

## Data Availability

The sequences utilized in this study were previously deposited in the NCBI SRA database under Bioproject numbers PRJNA317462 and PRJNA278367 by the CDC and PRJNA844269 by the University of Manitoba.

## SUPPLEMENTAL MATERIAL

Table S1 spectrum.01338-26-s0001.xlsx Differences in frequency between DGI and other niches.

Table S2 spectrum.01338-26-s0002.xlsx Population demographics and strain LIN codes.

ASM does not own the copyrights to Supplemental Material that may be linked to, or accessed through, an article. The authors have granted ASM a non-exclusive, world-wide license to publish the Supplemental Material files. Please contact the corresponding author directly for reuse.
